# Machine learning and network pharmacology identify keloid biomarkers (AMPH, TNFRSF9) and therapeutic targets (IL6, HAS2) for aloe-derived quercetin

**DOI:** 10.1371/journal.pone.0340960

**Published:** 2026-01-16

**Authors:** Congli Jia, Fu Yang, Yingchun Li

**Affiliations:** 1 Department of Plastic Surgery, Plastic Surgery Hospital of Shandong Second Medical University, Weifang, Shandong, China; 2 Department of Hepatobiliary Surgery, First Affiliated Hospital of Kunming Medical University, Kunming, Yunnan, China; 3 Weifang Traditional Chinese Medicine Industry Development Promotion Association, Weifang, Shandong, China; Shiraz University, IRAN, ISLAMIC REPUBLIC OF

## Abstract

**Objective:**

This study aimed to identify diagnostic biomarkers for keloid and explore potential therapeutic agents from traditional Chinese medicine (TCM) by integrating network pharmacology approaches. Specifically, we sought to uncover key molecular targets for Aloe vera and validate their roles in keloid pathogenesis.

**Methods:**

We integrated keloid transcriptome datasets (GSE218007 and GSE237752) by merging GEO data, and identifying differentially expressed genes (DEGs). Functional enrichment analysis (GO, GSEA) and machine learning approaches were applied to select diagnostic biomarkers. Candidate genes were validated via Receiver Operating Characteristic (ROC) curves in training and independent cohorts (GSE44270). PPI networks and Cytohubba algorithms identified hub genes, while TCMSP-screened compounds from Aloe vera were docked with targets using molecular docking.

**Results:**

91 Identified DEGs enriched in fibrosis-related pathways. Machine learning prioritized two diagnostic biomarkers: AMPH and TNFRSF9 (AUC > 0.85 in training/testing). PPI analysis revealed IL6 as a hub gene. Aloe vera-derived quercetin targeted HAS2 and IL6 (both P < 0.05 in validation), with molecular docking confirming stable binding (binding energy <−7 kcal/mol). IL6 emerged as both a key network hub and a therapeutic target, linking keloid and TCM mechanisms.

**Conclusion:**

AMPH and TNFRSF9 are promising diagnostic biomarkers for keloid, while quercetin from Aloe vera targets HAS2 and IL6, offering therapeutic potential. The dual role of IL6 underscores its centrality in keloid pathogenesis, connecting bioinformatics predictions with TCM pharmacology. This study provides a foundation for clinical prediction and targeted treatment strategies.

## 1. Introduction

Keloids represent a pathological outcome of aberrant cutaneous wound healing, characterized by fibroproliferative growths that progressively extend beyond the original wound boundaries [1,2]. Their morphological features and clinically aggressive behavior bear resemblance to non-malignant dermal neoplasms [3]. Although histologically benign, keloids frequently manifest clinically significant burdens, including persistent pain, pruritus and disfigurement, profoundly impacting patients’ quality of life [4,5]. Current therapeutic strategies remain suboptimal: surgical excision alone exhibits very high recurrence rates [6], while intralesional corticosteroid injections, the first-line intervention, demonstrate variable efficacy with inevitable recurrence [7]. Combination therapies (e.g., corticosteroids with 5-fluorouracil) show modest improvements but are limited by adverse effects such as intractable pain, cutaneous atrophy, pigmentary alterations and ulceration [8,9].

The high recurrence rates and iatrogenic complications associated with existing treatments, coupled with the invasive nature of diagnostic standards (e.g., histopathological biopsy), underscore the urgent need for two parallel advancements: (1) non-invasive biomarkers enabling early detection and risk stratification to guide preventive interventions. (2) Novel therapeutic agents targeting key pathogenic pathways while minimizing off-target effects. This dual approach is critical to addressing the unmet clinical demands in keloid management.

Machine learning (ML), as is increasingly being applied in bioinformatics research, demonstrating particular advantages in the screening of disease-related feature genes. For instance, researchers have utilized random forest and support vector machine algorithms to identify sphingolipid metabolism-related genes as potential therapeutic targets in keloid formation [10]. Compared with individual methods, the combination of LASSO (Least Absolute Shrinkage and Selection Operator) and SVM-RFE (Support Vector Machine-Recursive Feature Elimination) provides a more robust screening strategy, contributing to the development of more reliable predictive models [11,12]. This integrated machine-learning approach has been successfully employed to identify characteristic genes for various diseases [13,14]. TCM is one of the world’s oldest medical systems, in which natural substances with multiple chemical components have long been employed to treat various diseases [15]. Among these natural compounds, quercetin has attracted research attention due to its anti‐fibrotic properties, primarily mediated through the regulation of inflammatory responses [16]. However, the integration of machine learning with TCM to identify disease‐specific characteristic genes and explore potential therapeutic targets remains relatively underdeveloped. This gap is particularly evident in the context of keloid, where such interdisciplinary approaches could offer novel mechanistic and therapeutic insights.

Aloe vera, a medicinal plant with a long history of use, has been widely reported for its efficacy in wound healing and skin treatment [17]. It contains numerous bioactive compounds, such as quercetin and curcumin, which exhibit notable anti-inflammatory, antioxidant, and immunomodulatory properties [18]. Additionally, Aloe vera is commonly incorporated into cosmetic and skincare products due to its beneficial dermatological effects [19]. Given the role of chronic inflammation in the pathogenesis of keloid formation, we hypothesize that Aloe vera may have therapeutic potential in the treatment of keloids.

Furthermore, the complex and multifactorial pathogenesis of keloids presents a significant challenge for identifying key drivers and therapeutic interventions. Conventional single-target approaches have shown limited efficacy. Therefore, we employed a comprehensive analytical strategy integrating machine learning and network pharmacology. This approach is necessary to decipher the complex molecular interactions underlying keloid pathogenesis and to systematically identify critical diagnostic biomarkers and potential therapeutic targets from a network perspective. We further validate the binding affinities of its bioactive constituents to keloid-associated therapeutic targets through molecular docking simulations. This dual-strategy framework bridges phytochemical characterization with computational validation, offering mechanistic insights into how Aloe vera may modulate critical pathways in keloid suppression.

## 2. Methods

### 2.1. Data integration and preprocessing

#### 2.1.1. Dataset acquisition and merging.

The keloid datasets GSE218007 (comprising 23 keloid and 6 normal skin samples, platform GPL23126) and GSE237752 (comprising 3 keloid and 3 normal samples, platform GPL24676) were utilized as the training set. Dataset GSE44270 (comprising 9 keloid and 3 normal skin samples, platform GPL6244) served as an independent validation set. All samples were derived from human skin fibroblast mRNA. These datasets were obtained from the Gene Expression Omnibus (GEO) database (https://www.ncbi.nlm.nih.gov/) and are publicly available, no new patient data were generated for this study. Given that the two training datasets, GSE218007 and GSE237752, were generated using distinct microarray platforms (GPL23126 and GPL24676, respectively), a significant platform-specific batch effect was anticipated. To integrate these datasets and mitigate this non-biological variation, we employed a multi-step procedure using R (version 4.4.1) with key packages including limma and sva. First, the gene symbols common to both platforms were identified and extracted to create a unified gene list for subsequent analysis. The expression matrices from both datasets were then merged based on this set of common genes. To harmonize the data distributions across the merged dataset, we applied the ComBat algorithm from the sva package to adjust for location and scale shifts between platforms by estimating parameters for each feature within a batch and then shrinking these estimates towards the overall mean, thereby removing the systematic bias.

#### 2.1.2. Principal component analysis (PCA).

To evaluate batch correction efficacy by visualizing pre- and post-correction sample clustering Principal Component Analysis (PCA) was performed using R (version 4.4.1).

### 2.2. Differential expression analysis

#### 2.2.1. Identification of differentially expressed genes (DEGs).

Differentially Expressed Genes were identified with the screening criteria as follows: Adjusted P-value (adj.P.Val) < 0.05, Absolute log2 fold change (|log2FC|) > 1.

#### 2.2.2. Functional enrichment analysis.

To functionally characterize the DEGs, Gene Ontology (GO) and Gene Set Enrichment Analysis (GSEA) were carried out utilizing R (version 4.4.1). The significantly enriched terms and pathways were subsequently filtered using an adjusted P-value＜0.05.

### 2.3. Machine learning-based biomarker selection

#### 2.3.1. Univariate logistic regression pre-screening.

A univariate logistic regression analysis was employed as an initial step to pre-filter genes exhibiting statistically significant associations with the phenotypic outcome (keloid vs. normal skin). In this analysis, each gene’s expression level was included as the sole independent variable in a separate regression model. Genes with a p-value < 0.05 from the univariate logistic regression model were deemed statistically significant and advanced to subsequent analysis.

#### 2.3.2. Multi algorithm feature selection.

The LASSO regression was implemented using the glmnetpackage (version 4.4) in R. To fit the model, a 10-fold cross-validation was conducted (nfolds = 10) to determine the optimal value of the regularization parameter, lambda (λ). The specific λ value that yielded the minimum mean squared error (MSE) across the cross-validation folds was selected to finalize the model, ensuring an optimal balance between bias and variance [20].

The Random Forest algorithm was executed using the randomForest package in R. An ensemble of 500 decision trees (ntree = 500) was grown to enhance predictive accuracy and stability. A key output of the Random Forest model is a metric of variable importance, which quantifies the contribution of each feature to the model’s predictive power. Based on this importance score, the top 15 genes that exerted the greatest influence on the model were retained for subsequent analysis [21].

A Recursive Feature Elimination (RFE) process was coupled with a Support Vector Machine (SVM) classifier to identify a minimal subset of non-redundant features that maintained high classification performance using R package “e1071”. This RFE-SVM framework was evaluated using a robust 5-fold cross-validation strategy (k = 5). The process iteratively pruned the least important features based on the SVM model’s criteria. The optimal feature subset was selected by applying a dual-threshold criterion: it had to achieve a peak classification accuracy exceeding 85% while simultaneously maintaining a MSE below 0.15, ensuring a feature set that is both discriminative and parsimonious [22].

#### 2.3.3. Intersection genes and validation.

Genes overlapping across LASSO, random forest and SVM results were identified. To independently validate the discriminative capacity of the feature genes, their expression was first examined in the testing cohort GSE44270 (9 keloid and 3 normal skin samples). Subsequently, the diagnostic efficacy of these genes was evaluated by constructing ROC curves in both the combined training set (total n = 35; 26 keloid, 9 normal) and the independent validation set GSE44270 (n = 12; 9 keloid and 3 normal). The Area Under the Curve (AUC) for each gene served as a metric for classification performance.

### 2.4. Protein-protein interaction (PPI) network analysis and hub genes selection

DEGs were submitted to STRING (https://cn.string-db.org) [23] to conduct PPI Network (confidence score threshold > 0.4; disconnected nodes hidden). The results were visualized using Cytoscape (version 3.10.3) [24] and six hub genes were systematically identified through the CytoHubba plugin by integrating three centrality metrics: Closeness centrality, Degree centrality and Edge Percolated Component (EPC). The EPC algorithm identifies key connected components that emerge at different density thresholds. A commonly used approach is to select the component that maximizes a specific metric, which is often a function of the component’s size and the average weight of the edges within it. This helps pinpoint the most significant and coherent functional module in the network. These computationally prioritized hub genes, representing critical nodes in keloid pathogenesis, have been archived in Supplementary Material S1 Table for reproducibility.

### 2.5. Targets of herbal medicine Aloe vera prediction and validation

#### 2.5.1. Bioactive compound screening and standardization.

Bioactive constituents of Aloe vera were systematically screened from the Traditional Chinese Medicine Systems Pharmacology (TCMSP) database (https://old.tcmsp-e.com) using Oral bioavailability (OB) ≥30% and Drug-likeness (DL) ≥0.18 thresholds. Corresponding targets were retrieved and unified via UniProt (https://www.uniprot.org). Details of Aloe vera ingredients and Aloe vera Targets symbol were documented in Supplementary Material S2 and S3 Table.

#### 2.5.2. Targets intersection and expression validation.

Overlapping genes between Aloe vera targets and keloid DEGs were identified. To validate the differential expression patterns of the overlapping target genes, we performed an independent analysis using the validation dataset GSE44270 with significance thresholds set at Adjusted P-value < 0.05 and |log2 fold change| > 1 to ensure biological relevance.

### 2.6. Molecular docking validation

#### 2.6.1. Ligand and receptor preparation.

To identify the bioactive compounds targeting the candidate proteins, we analyzed the Aloe vera Targets symbol file (available in Supplementary Material S3 Table) and determined that quercetin was the common active component targeting both HAS2 and IL6. The SMILES notation and 3D structure of quercetin (PubChem CID: 5280343) were retrieved from the PubChem database (https://pubchem.ncbi.nlm.nih.gov). For the target proteins, the tertiary structures of HAS2 (UniProt ID: Q92819) and IL6 (UniProt ID: P05231) were obtained from the AlphaFold Protein Structure Database (https://alphafold.ebi.ac.uk).

#### 2.6.2. Docking of quercetin and targets.

The protein structure was prepared using PyMOL (version 2.4) by removing water molecules and adding hydrogen atoms. Docking simulations of quercetin into the proteins IL6 (P05231) and HAS2 (Q92819) were executed with AutoDock Vina 1.1.2 using a maximally sized grid box to facilitate blind docking; all other parameters remained default [25]. The top 10 poses generated for each protein were evaluated, and the pose exhibiting the lowest binding energy and highest cluster membership was identified as the most stable binding mode. Subsequent visualization and interaction analysis were performed using PyMOL 2.4 and Discovery Studio 2019.

## 3. Results

### 3.1. Batch effect analysis using principal component analysis (PCA)

**Fig 1:**
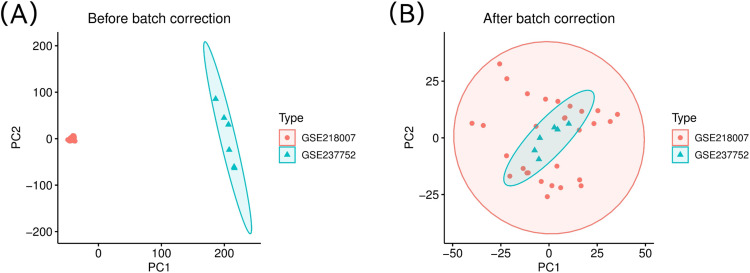
PCA of GSE218007 and GSE237752 datasets before and after batch effect adjustment. (A) PCA prior to batch effect adjustment. The plot shows a clear separation of samples from the two datasets (GSE218007, red circles; GSE237752, cyan triangles) along the first principal component. (B) PCA following batch effect adjustment. After correction, the samples from both datasets show substantial overlap.

Prior to batch effect adjustment, principal component analysis revealed substantial dispersion between the GSE218007 and GSE237752 datasets along both principal components 1 (PC1) and PC2, indicating a strong batch effect. Each dataset cluster is encircled with a corresponding colored ellipse (Fig 1A). The samples clustered predominantly by their dataset origin rather than biological phenotype (keloid versus control), demonstrating that batch effects constituted the primary source of variation in the raw data. Following batch effect correction, a marked improvement in data integration was observed. Post-correction PCA demonstrated that samples from both datasets showed appropriate clustering according to biological phenotype rather than study origin (Fig 1B).

### 3.2. Identification and functional enrichment analysis of DEGs

**Fig 2:**
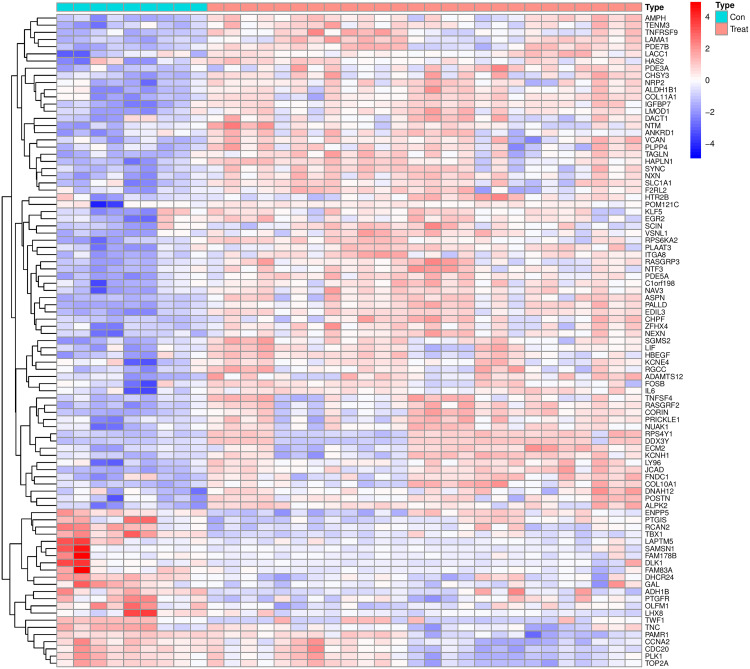
Heatmap of DEGs. The color scale represents normalized expression levels, with red indicating high expression and blue indicating low expression.

**Fig 3:**
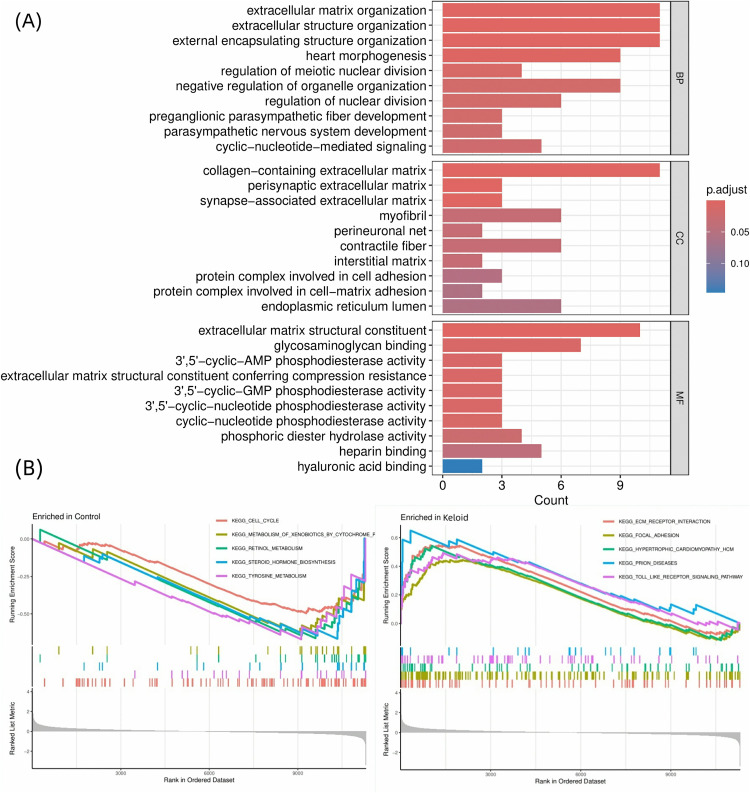
Enrichment analysis of DEGs. (A) GO enrichment analysis. The bar plots show the significant enrichment terms for the DEGs across the three GO categories: Biological Process (BP), Cellular Component (CC), and Molecular Function (MF). The length of the bar represents the number of genes associated with each term, and the color corresponds to the statistical significance, with red indicating greater significance. (B) GSEA enrichment plots. The plots illustrate the enrichment of representative KEGG pathways that are significantly enriched in the control (left) and keloid (right) groups.

Transcriptomic analysis identified 91 DEGs between keloid lesions and normal skin, with hierarchical clustering revealing distinct expression patterns between groups (Fig 2). GO enrichment analysis demonstrated significant overrepresentation of extracellular matrix (ECM)-related biological processes and cellular components among the DEGs (Adjusted P-value < 0.05). Key enriched terms included: extracellular matrix organization, extracellular structure organization, external encapsulating structure organization, collagen−containing extracellular matrix and extracellular matrix structural constituent (Fig 3A). GSEA further corroborated these findings, showing significant positive enrichment of ECM-related pathways in keloid tissues, including: ECM-receptor interaction, Focal adhesion and Toll-like receptor signaling pathway (Fig 3B). The significantly enriched biological processes and signaling pathways are intricately associated with, and in many cases directly orchestrate, key pathological events in keloid formation, such as aberrant extracellular matrix deposition, dysregulated fibroblast proliferation, and sustained inflammatory responses. This compelling association further underscores the potential of the corresponding differentially expressed genes to serve as promising therapeutic targets.

### 3.3. Feature genes screening based on machine learning

#### 3.3.1. LASSO regression.

**Fig 4:**
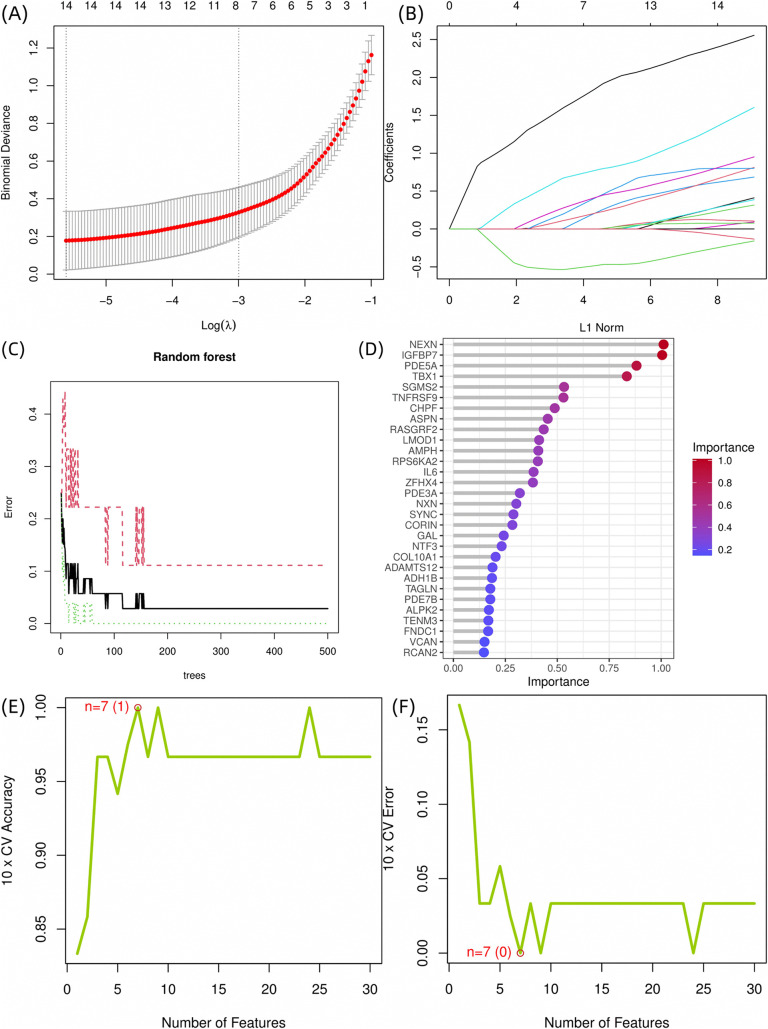
Machine learning workflow for feature gene selection and model performance evaluation. (A) Identification of the optimal regularization parameter (λ) using cross-validation. The binomial deviance (red curve) across values of log(λ) is plotted, with the gray band representing the confidence interval. (B) LASSO coefficient shrinkage paths. As regularization intensifies, coefficients for less relevant features are shrunk to zero, with 14 genes retaining non-zero coefficients at the optimal threshold. (C) Random Forest Out-of-Bag (OOB) Error Convergence. The plot depicts the decline in OOB error rate as the number of trees increases. (D) Variable Importance Based on MeanDecreaseGini. Genes are ranked by their importance scores, with longer bars indicating greater contribution to classifying keloid versus normal samples. (E) SVM-RFE feature selection accuracy curve. The plot shows the 10-fold cross-validation (CV) accuracy versus the number of selected features. (F) SVM-RFE cross-validation error profile. The relationship between feature subset size and 10-fold CV classification error is displayed.

Variable selection was performed using LASSO regression. The cvfit curve (Fig 4A) identified optimal regularization at λ, achieving 85% cross-validated accuracy while retaining parsimony. Corresponding coefficient paths (Fig 4B) demonstrated progressive feature elimination, ultimately selecting 14 non-zero coefficient genes at this threshold.

#### 3.3.2. Random Forest.

The random forest classification model demonstrated robust predictive performance, achieving an out-of-bag (OOB) error rate of <15% (Fig 4C), indicating excellent discriminative capacity between keloid and normal tissue samples. The OOB error is an internally estimated generalization error calculated using the data points not sampled in the bootstrap process for building each individual tree. This provides an unbiased evaluation of the model’s performance on unseen data without the need for a separate test set, and a lower OOB error rate signifies stronger predictive ability. Variable importance analysis, assessed through MeanDecreaseGini identified multiple genes with substantial predictive value (importance score >0.2, Fig 4D). From these, we selected the top 15 most influential genes (ranked by importance scores) as candidate biomarkers for subsequent validation.

#### 3.3.3. RFE-SVM.

The SVM-RFE feature selection curve (Fig 4E) revealed a monotonic increase in accuracy to 100% at cardinality k = 7, followed by plateauing and a corresponding error rate minimization to 0% at identical k (Fig 4F). This cross validation confirmed k = 7 as the optimal feature number, yielding the final feature genes.

### 3.4. Intersection genes and validation

**Fig 5:**
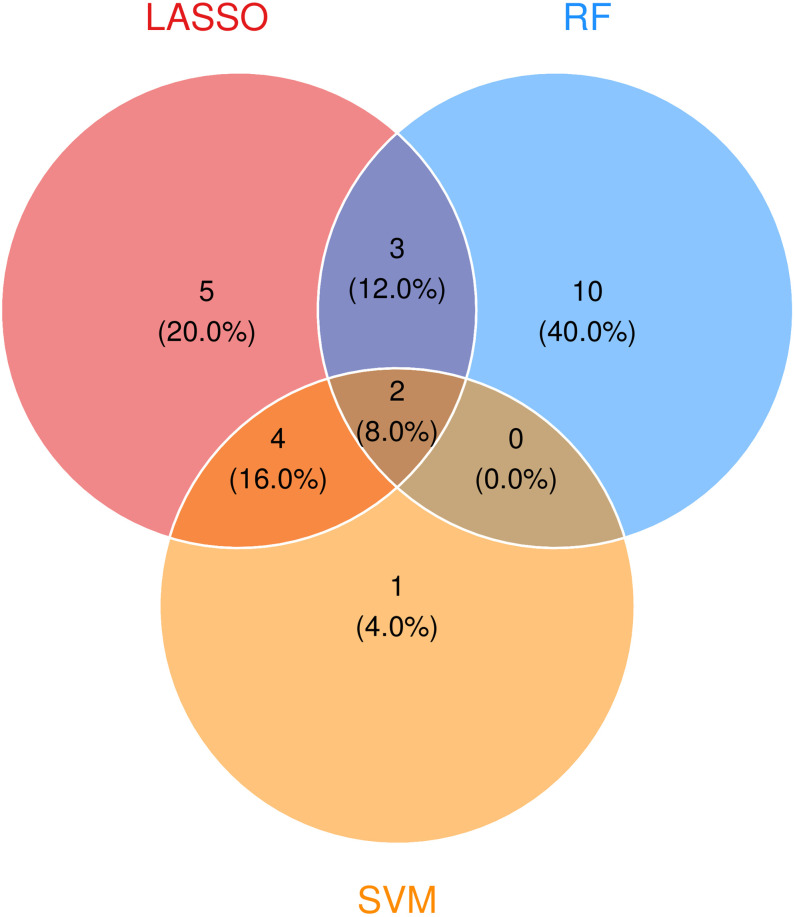
Identification of consensus biomarker genes for keloid diagnosis. Consensus feature gene selection by LASSO, Random Forest, and SVM-RFE.

**Fig 6:**
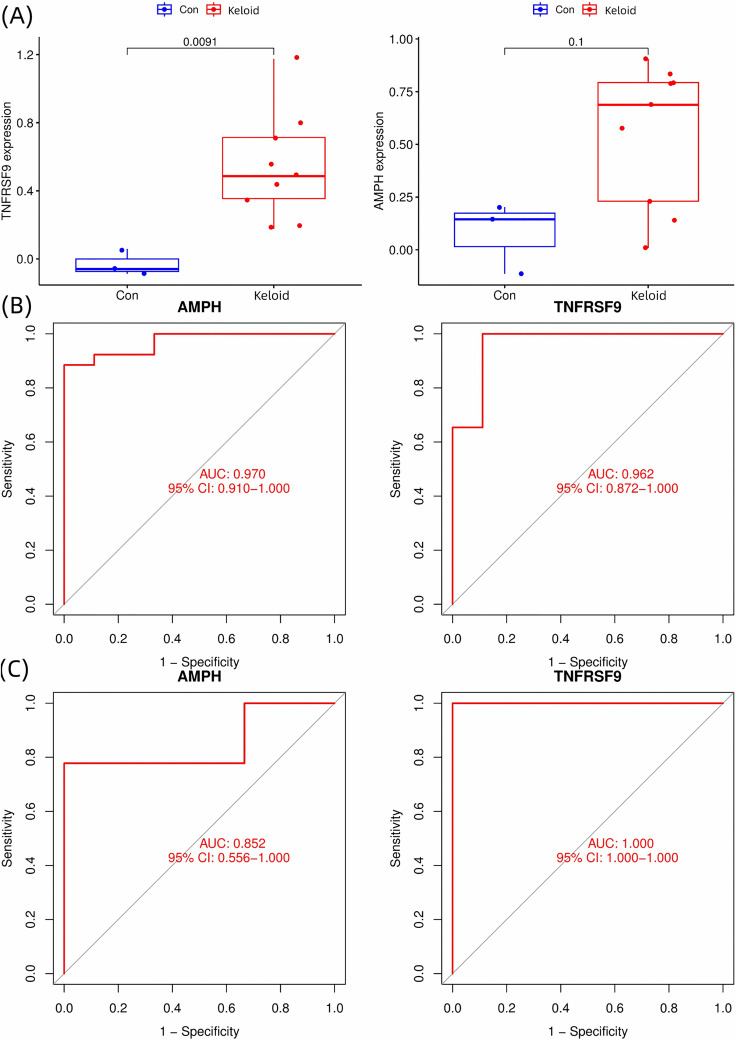
Validation of feature genes. (A) Validation of candidate gene expression in the testing cohort GSE44270 (n = 12; 9 keloid and 3 normal). Box plots comparing the expression levels of the two consensus biomarkers, (left) TNFRSF9 and (right) AMPH, between Keloid (red) and Control (blue) samples. (B) ROC curve for features in the merged training cohort (total n = 35; 26 keloid, 9 normal). (C) ROC curve for features in the testing cohort GSE44270 (n = 12; 9 keloid and 3 normal).

The intersection of feature genes derived from LASSO, random forest and SVM-RFE yielded two consensus biomarkers: AMPH and TNFRSF9 (Fig 5). While both genes showed elevated expression in keloids (log2FC > 1), only TNFRSF9 reached statistical significance (p = 0.0091 vs p = 0.1 for AMPH) in the testing cohort GSE44270 (n = 12; 9 keloid and 3 normal) (Fig 6A). Notably, despite AMPH’s p-value＞0.05, its diagnostic performance remained strong (AUC > 0.85 in both training and testing cohorts) and the AUC of TNFRSF9 is 0.962 and 1.0 in training cohort (Fig 6B) and testing cohort (Fig 6C) respectively, suggesting clinical utility may persist despite imperfect statistical significance in this validation cohort.

### 3.5. PPI network analysis and hub genes selection

**Fig 7:**
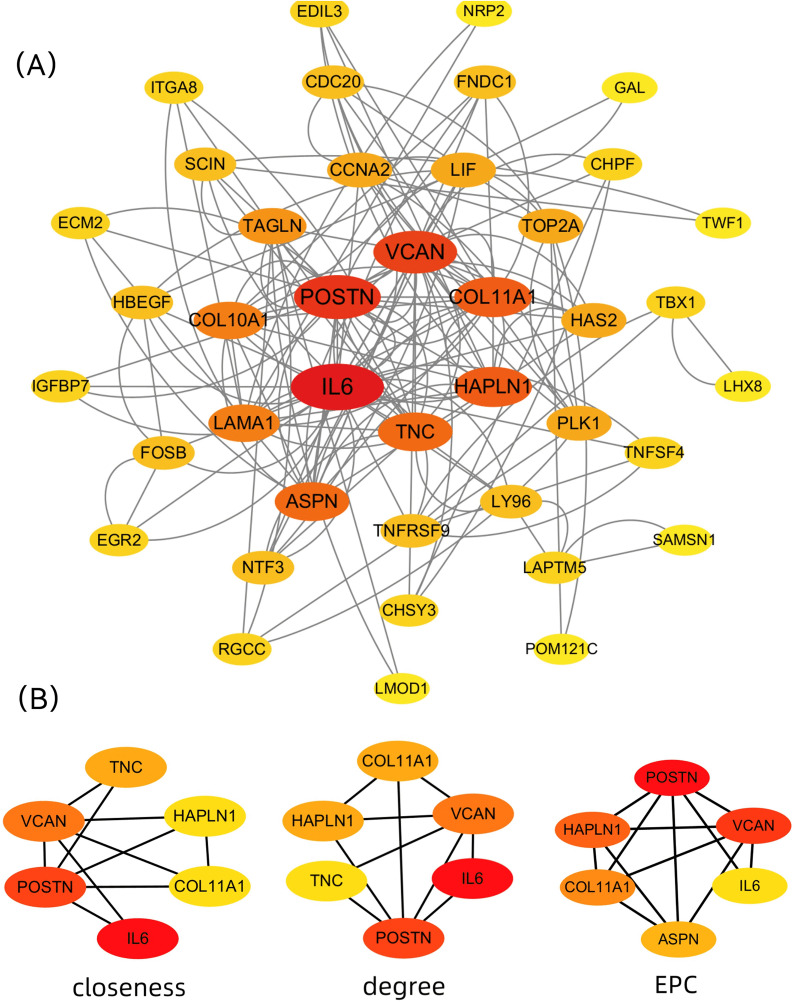
PPI network construction and hub gene identification. (A) PPI network of DEGs. The network depicts interactions among keloid-related DEGs, with nodes representing proteins and edges representing interactions. (B) Topological analysis of hub genes using CytoHubba. The core hub genes were consistently identified by three distinct centrality algorithms: Closeness, Degree, and EPC which is a connected subgraph formed through a process called edge percolation, where edges in a network are probabilistically selected based on a specific criterion, such as a weight or confidence score.

Analysis of the PPI network (Fig 7A) revealed the interaction between DEGs. Subsequent network topology analysis through CytoHubba identified six hub genes (IL6, POSTN, VCAN, COL11A1, HAPLN1, TNC) via three methods of calculation respectively (Closeness, Degree and EPC) (Fig 7B), representing key regulatory nodes in the keloid. Details were archived in Supplementary Material S1 Table. This high degree of connectivity among hubs implies they may function in a coordinated manner, potentially coregulating key pathological events in keloid pathogenesis.

### 3.6. Acquisition and validation of Aloe vera targets for keloid

**Fig 8:**
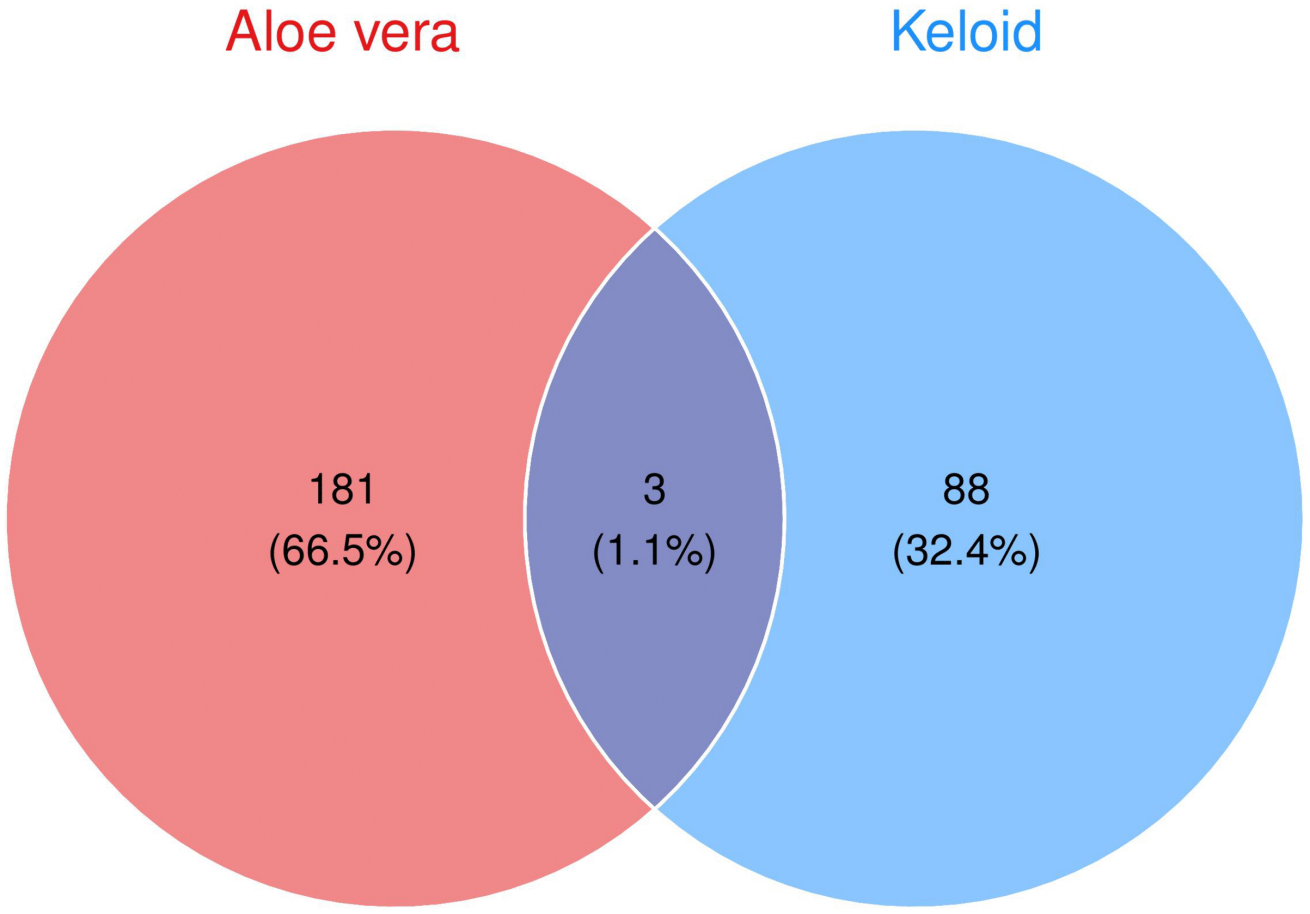
Identification and of Aloe vera targets for keloid. Identification of overlapping targets between Aloe vera and keloid.

**Fig 9:**
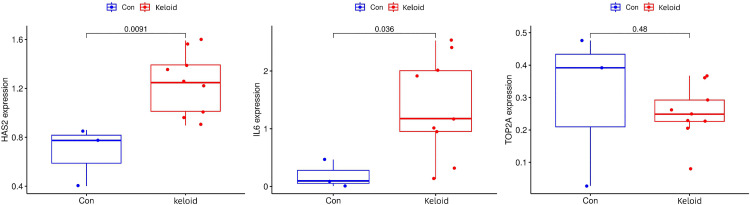
Validation of Aloe vera targets for keloid. Validation of candidate gene expression in an independent cohort GSE44270 (n = 12; 9 keloid and 3 normal). Box plots comparing the expression levels of HAS2, IL6, and TOP2A between keloid and normal skin (Con) samples in the independent validation cohort GSE44270 (n = 12; 9 keloid and 3 normal).

Details of Aloe vera ingredients and Aloe vera targets symbol derived from TCMSP database were documented in Supplementary Material S2 and S3 Tables. Three intersection genes of Aloe vera targets and keloid DEGs were obtained (Fig 8). Quantitative analysis of target gene expression in the independent validation cohort confirmed significant upregulation of both HAS2 (P = 0.0091) and IL6 (P = 0.036), while TOP2A failed to reach statistical significance (P = 0.48) (Fig 9). These results strongly implicate HAS2 and IL6, but not TOP2A, as the primary molecular targets mediating Aloe vera’s anti-keloid effects.

### 3.7. Molecular docking validation

**Fig 10:**
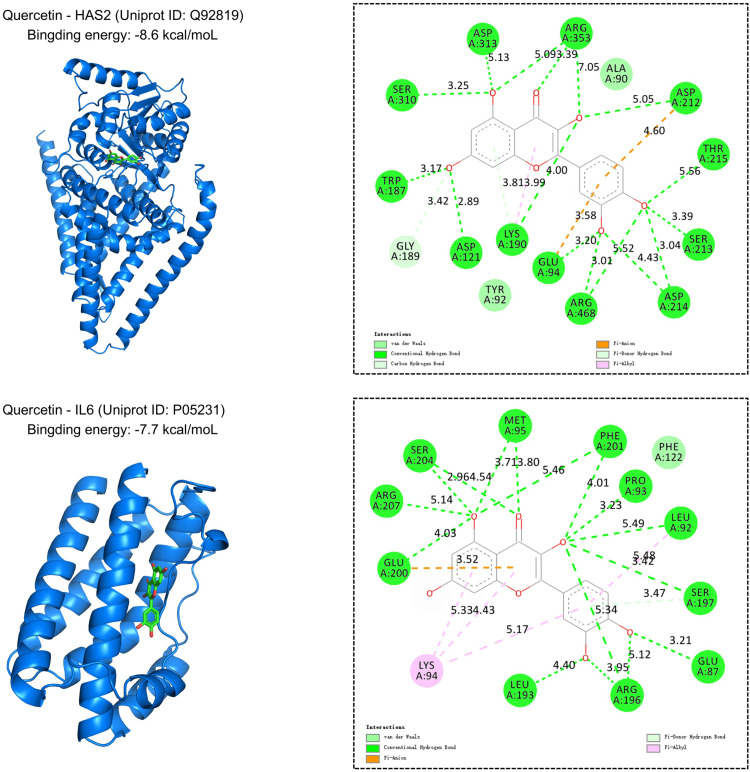
Molecular docking analysis of quercetin with key keloid-related targets. The 3D structure (left) shows quercetin (green sticks) embedded in the binding pocket of HAS2 or IL6, with a calculated binding energy of −8.6 kcal/mol and −7.7 kcal/mol respectively. The 2D interaction map (right) details hydrogen bonds (green dashed lines) and hydrophobic interactions (purple dashed lines) with key residues.

Molecular docking revealed that quercetin, the active component of Aloe vera (SMILES: C1 = CC(=C(C = C1C2=C(C(=O)C3 = C(C = C(C = C3O2)O)O)O)O)O), demonstrated stable binding interactions with two key target proteins HAS2 and IL6. Quercetin is primarily stabilized within the binding pocket of HAS2 through an extensive hydrogen-bonding network involving residues ASP A:310 and ARG A:468 which serve as critical anchoring points, positioning the ligand near the protein surface and contributing to the favorable binding energy of −8.6 kcal/mol. In the IL6 binding site, quercetin penetrates deeper into the protein core, forming key hydrogen bonds with GLU A:200, SER A:204, ARG A:207 and other residues, The residues GLU A:200 and ARG A:207 are particularly significant in orienting the ligand within the hydrophobic cavity, supporting a binding energy of −7.7 kcal/mol (Fig 10).

The potent binding of quercetin to HAS2 and IL6, as evidenced by low binding energies, indicates a targeted interaction. This interaction likely inhibits keloid pathogenesis by either directly repressing gene expression or, more plausibly, by altering protein conformation to obstruct their functional activity in critical signaling pathways.

## 4. Discussion

In our study, DEGs between keloids and normal skin tissues were significantly enriched in ECM-related biological processes and cellular components via GO analysis (Fig 3A). GSEA further revealed prominent enrichment of DEGs in ECM-related signaling pathways (Fig 3B). Prior studies have established that the TGF-β/Smad signaling pathway drives fibroblast overactivation and collagen deposition in keloids.

Through machine learning-based feature selection, two feature genes—AMPH (Amphiphysin) and TNFRSF9 (Tumor Necrosis Factor Receptor Superfamily Member 9)—were identified as potential keloid biomarkers. The AMPH-encoded protein, a member of the membrane-associated protein family, is primarily implicated in membrane morphogenesis, clathrin-mediated endocytosis and synaptic vesicle recycling [26]. We hypothesize that AMPH, by participating in clathrin-mediated endocytosis (CME), may influence the internalization trafficking and signaling duration of the TGF-β receptor (TβR). This could promote TβR entry into signal-active early endosomes, prolonging SMAD-mediated signaling and exacerbating the pro-fibrotic response [27]. Furthermore, AMPH might regulate the nucleocytoplasmic translocation efficiency or stability of SMAD proteins via its role in endocytosis, potentially establishing a positive feedback loop with TGF-β signaling that synergistically amplifies pro-fibrotic signals and leads to excessive deposition of extracellular matrix. Notably, AMPH may also facilitate the endocytosis of inflammatory receptors, enhancing NF-κB signaling and leading to sustained release of pro-inflammatory cytokines (e.g., TNF-α, IL-6). This would contribute to a persistent pathological inflammatory microenvironment, which in turn stimulates fibroblast activation and supports fibrosis progression.

TNFRSF9, a member of the TNF receptor superfamily (TNFRSF), encodes the transmembrane protein CD137/4–1BB [28], a critical immunoregulatory molecule predominantly expressed on activated CD8 + T cells and natural killer (NK) cells [29]. Previous evidence suggested that TGF-β1 negatively regulated the co-stimulatory checkpoint 4–1BB/TRAF1 axis in chronic viral infections [30,31]. We hypothesize that 4−1BB (TNFRSF9) signaling might activate the NF-κB pathway [32], which synergizes with TGF-β1/Smad signaling. This interplay is predicted to perturb the TGF-β1 negative feedback regulation, leading to its sustained activation and the subsequent upregulation of pro-fibrotic gene expression.

In our study, AMPH did not reach statistical significance in validation (P = 0.1), which may reflect the small cohort size or residual batch effects. However, the robust discriminative power of these genes was corroborated by ROC analysis. The TNFRSF9 biomarker achieved an AUC of 1.00 in the validation set, indicating perfect separability between classes based on the selected features and model configuration. While an AUC of 1.0 represents ideal performance on the available data, we acknowledge that this result should be interpreted with caution due to the relatively limited sample size of the GEO microarray dataset. In learning, especially with high-dimensional data, such performance can sometimes reflect overfitting, where the model learns noise or dataset-specific variations rather than generalizable biological patterns. Nonetheless, this outcome underscores the strong discriminatory potential of TNFRSF9 in keloid pathogenesis within the constraints of the current dataset. Further validation in larger, independent cohorts is essential to confirm its robustness and clinical applicability. These findings may position AMPH and TNFRSF9 as promising non-invasive biomarkers for keloid risk stratification. Their strong discriminative capacity may facilitate the identification of high-risk individuals, enabling early clinical interventions. Future studies should prioritize: 1. Functional validation to delineate their roles in TGF-β1-driven fibrogenesis and immune modulation. 2. Multi-center validation with expanded cohorts to confirm diagnostic reproducibility.

Clinical management of keloids remains challenging due to high recurrence rates and toxic and side effects associated with existing pharmacological agents. The therapeutic potential of Aloe vera in keloid remains underexplored. Given the inherent advantages of natural compound—including its favorable safety profile, accessibility, and cost-effectiveness—as well as its documented applications in skin protection, this study identified shared targets with keloid pathology through network pharmacology. By constructing a “compound-target-pathway” axis, we aim to elucidate their potential multi-modal mechanisms in suppressing fibrosis, inflammation, and the dysregulated activation of fibroblasts.

Quercetin, a dietary bioflavonoid and potent antioxidant [33,34], has been shown to exhibit superior antifibrotic efficacy compared to vitamin E in a bleomycin-induced rat model of pulmonary fibrosis [16]. This enhanced activity is attributed to its dual capacity to restore pulmonary redox homeostasis and suppress the inflammatory cascade [35].

In our investigation, IL6 and HAS2 were identified as quercetin’s anti-keloid targets through network pharmacology. Notably, IL6 emerged as a central hub within the keloid-associated PPI network, implicating its pivotal role in keloid pathogenesis (Fig 7).

Elevated IL6 levels have been consistently reported in keloid tissues [36,37], with studies confirming increased IL6 secretion by cultured keloid fibroblasts into conditioned media [38]. Treatment of normal fibroblasts with exogenous IL6 resulted in an increase in actin and ECM alignment to levels close to those observed in keloid fibroblasts [38]. While TGF-β-dependent myofibroblast differentiation remains a canonical driver of fibrogenesis, some research data indicated pathological ECM remodeling arised from IL6-mediated autocrine signaling cascades [39]. For instance, in systemic sclerosis (SSc), IL6 inhibition reduces dermal thickening and downregulates fibrotic markers (e.g., α-smooth muscle actin) by suppressing the TGF-β/STAT3 axis [39,40]. Mechanistically, IL6 directly activates dermal fibroblasts, exacerbating ECM overproduction and tissue stiffening [41]. Previous studies in SSc murine models further validate that IL6 blockade attenuates dermal fibrosis, suggesting pleiotropic roles of IL6 in both initiation and perpetuation of fibrotic cascades [40,41].

Quercetin has been demonstrated to significantly attenuate the production of pro-inflammatory cytokines, including TNF-α, IL-1β, and IL6, in LPS-stimulated RAW264.7 macrophages by inhibiting the NF-κB pathway [42]. Molecular docking analysis indicates that quercetin binds stably to IL6, suggesting that this interaction may disrupt downstream signaling cascades, such as the TGF-β/STAT3 axis and the NF-κB pathway. Consequently, these actions are proposed to reduce inflammation, enhance antioxidant defenses, and ameliorate pathological ECM deposition, collectively contributing to its antifibrotic effects. This indicates that quercetin might emerge as a promising anti-keloid strategy by mechanistically disentangling IL6-induced ECM dysregulation from the TGF-β-mediated fibrotic cascade.

Hyaluronan Synthase 2 (HAS2) catalyzes the synthesis of hyaluronan (HA), a major component of the ECM. Notably, low-molecular-weight HA (LMW-HA), the predominant HA isoform in fibrotic livers, can activate CD44, Toll-like receptor 4 (TLR4) and Notch signaling pathways, exhibiting pro-inflammatory and pro-fibrotic properties [43]. Overexpression of HAS2 in fibroblasts promotes an aggressive phenotype, leading to severe fibrosis [44]. GSEA revealed that the DEGs in keloids were significantly enriched in the toll-like receptor (TLR) signaling pathway. This finding suggests that the upregulated expression of HAS2 in keloids may promote hyaluronic acid (HA) secretion, which in turn could potentially activate the TLR and Notch signaling pathways, thereby contributing to their pro-inflammatory and pro-fibrotic characteristics. In addition, TGF-β transcriptionally activates HAS2 to stimulate HA biosynthesis, which in turn modulates TGF-β-induced pulmonary fibroblast-to-myofibroblast differentiation and collagen deposition [45]. We suppose that disrupting this circuit at any node-for instance, suppressing HAS2-mediated HA production-could extinct TGF-β-driven myofibroblast transdifferentiation and collagen hyperaccumulation. Compelling evidence demonstrated that HAS2 depletion significantly suppressed the proliferation of fibrogenic fibroblasts while inducing G1 phase cell cycle arrest, mechanistically linking HAS2 inhibition to proliferative quiescence via cell cycle regulation [46]. The stable binding of quercetin to HAS2 implies that it may disrupt the above hypothesized regulatory loop by targeting HAS2 inhibition, indirectly inhibit TGF-β → HAS2 → HA → TLR axis-mediated ECM deposition and also inhibit fibroblast proliferation by regulating the cell cycle, thereby improving keloid.

It is important to clarify the distinction between the diagnostic biomarkers (AMPH, TNFRSF9) and the therapeutic targets (IL6, HAS2) identified in this study. The former were derived from a machine learning analysis of keloid transcriptomic data, prioritizing genes with the highest predictive power for disease classification. In contrast, the potential therapeutic targets were identified through a network pharmacology approach focusing on the intersection between Aloe vera and keloid pathogenesis, which emphasizes pharmacological tractability. This methodological difference explains the non-overlap between the two gene sets, as they serve complementary but distinct purposes: one for diagnostic precision and the other for therapeutic exploration. However, the specific mechanisms of the targeted effects of quercetin and IL6/HAS2 in the treatment of keloids still need to be further explored.

This study has several limitations that warrant consideration. First, the precise biological roles of the identified biomarkers (AMPH/TNFRSF9) and therapeutic targets (IL6/HAS2) in keloid pathogenesis remain unvalidated through functional experiments; Second, the statistical power of our validation cohort may be constrained by its limited sample size; Third, while the diagnostic biomarkers demonstrate tissue-level specificity, their clinical utility requires further verification in non-invasive biofluids (e.g., serum) to assess detectability and correlation with disease severity. Finally, the translational application of quercetin via topical delivery necessitates optimization of physicochemical properties and cutaneous permeability-critical determinants of local bioavailability and therapeutic efficacy. In addition, we acknowledge the imbalance in sample sizes between the keloid and control groups within the utilized GEO datasets, where keloid samples outnumbered the controls. Such an imbalance has the potential to introduce bias and affect the generalizability of bioinformatics findings, as it may impact the estimation of variance and the power of statistical comparisons. To mitigate this, we employed analytical methods that are relatively robust to such disparities. Furthermore, the gene expression differences observed between groups were substantial and statistically significant, which provides confidence in the core findings regarding differentially expressed genes. Key future directions include using qPCR and Western blot to confirm their expression levels, and functional assays in keloid-derived fibroblasts to elucidate their roles in pathogenesis.

## 5. Conclusion

In conclusion, this study integrates machine learning-driven biomarker discovery with network pharmacology to unravel novel diagnostic and therapeutic avenues for keloid management. Mechanistically, we identify quercetin as a multi-target agent capable of simultaneously suppressing IL6-mediated autocrine signaling and disrupting a critical node (HAS2) within the self-reinforcing TGF-β/ HA regulatory circuit. This dual inhibition effectively attenuates the pathological ECM remodeling central to keloid progression. Our findings not only elucidate a promising phytochemical strategy for keloid intervention but also provide a scalable computational-experimental paradigm for unraveling complex fibrotic diseases.

Future studies should prioritize experimental validation of AMPH/TNFRSF9 in larger cohorts, functional characterization of IL6/HAS2 in keloid models and development of quercetin topical formulations to address the clinical need for safe and effective anti-keloid treatment.

## Supporting information

S1 Table Top 6 genes in PPI based on Degree, EPC and Closeness algorithm. This table presents the results of all genes in the PPI network analyzed using the Degree, EPC, and Closeness algorithms. The top six genes listed are identified as the core genes.
(XLSX)

S2 Table Aloe vera ingredients. This table presents relevant data on the primary chemical ingredients of Aloe vera, including MOL ID, molecular name, OB, and DL.
(XLSX)

S3 Table Symbol of Aloe vera Targets. This table presents the full names and corresponding standard symbols of the targets associated with the primary chemical constituents of Aloe vera.
(XLSX)
